# Adverse drug reactions of Highly Active Antiretroviral Therapy (HAART) in HIV infected patients at the General Hospital, Douala, Cameroon: a cross sectional study

**Published:** 2012-07-27

**Authors:** Henry Namme Luma, Marie-Solange Doualla, Simeon-Pierre Choukem, Elvis Temfack, Gloria Ashuntantang, Henry Achu Joko, Sinata Koulla-Shiro

**Affiliations:** 1 Department of Internal Medicine, Douala General Hospital, Douala, Cameroon; 2 Faculty of Medicine and Biomedical Sciences, University of Yaoundé 1, Yaoundé, Cameroon; 3 Depatment of Clinical Sciences, Faculty of Health Sciences, University of Buea, Buea, Cameroon; 4 Department of Internal Medicine, Yaoundé General Hospital, Yaoundé, Cameroon

**Keywords:** Adverse drug reaction, HAART, HIV

## Abstract

**Background:**

The use of highly active antiretroviral therapy (HAART) as the main option for management of people living with Human Immune deficiency virus (HIV) is associated with decrease morbidity and mortality. This is due to its effectiveness in inhibiting viral replication. However this effectiveness is not without adverse drug effects which in many settings are not monitored.

**Methods:**

A cross sectional clinical chart review of adult Cameroonian patients on HAART between 2003 and 2009 at the Douala General Hospital was done in search of reported HAART-associated Adverse Drug effects (ADRs). The prevalence of ADR defined as the proportion of the study population with ADR was determined and stratified by age, sex, weight and HAART regimen.

**Results:**

Sixty-six (19.5%) of the 339 patients on HAART reported ADRs. Among those who reported ADRs, 29.6% were on D4T-3TC-EFV, 29.3% on D4T-3TC-NVP, 16% on AZT-3TC-EFV and 10.8% on AZT-3TC-NVP. Peripheral Neuropathy was the most common ADR and represented 21.2% of all ADRs. Patients on D4T containing regimens were more likely to develop ADR (OR = 3.5, 95% CI 1.5 – 9.8, p<0.01) and 56.1% of all ADRs were associated to D4T. Hospital admissions were for patients with severe anaemia, no fatal cases of ADRs were recorded.

**Conclusion:**

HAART-associated ADRs are common and therefore should be actively looked for by caregivers so as to ameliorate the quality of life of HIV patients on treatment.

## Background

The introduction of highly active antiretroviral therapy (HAART) in developed countries in the late 90s has been associated with a remarkable decrease in AIDS-related mortality. This decrease in mortality has changed the perspective of HIV infection from that of a rapidly fatal to a chronic manageable infection [1]. Clinical benefits of HAART are due to its effectiveness in decreasing disease progression in HIV infected patients by sustained suppression of viral replication [2]. These clinical benefits however are not without unwanted effects called adverse drug reactions (ADRs). An ADR is defined as an appreciably harmful or unpleasant reaction, resulting from an intervention related to the use of a medicinal product (which in this case is HAART), which predicts hazard from future administration and warrants prevention or specific treatment, or alteration of the dosage regimen, or withdrawal of the product [3]. ADRs have been one of the most important limiting factors to the success of HAART [4] because they are responsible for new co-morbidities noticeable by the patients or their families and may result in decreased adherence to treatment which consequently might lead to virological failure [4] and poor prognosis. ADRs due to continuous exposure to antiretroviral drugs leave the caregiver with few options: decreasing the dosage of antiretroviral drugs thus compromising efficacy, withdrawing the offending drug and substituting it with another drug, or symptomatically treating the ADR. However, substituting the offending drug by the caregiver is difficult especially in resource limited settings because most HAART regimens exist as fixed dose combinations (FDC) of different drugs most of which are first line drugs with high toxicity profiles. The advent of new generation drugs with relatively low toxicity into the antiretroviral armamentarium is some hope that deleterious effects of HAART related ADRs in HIV patients would decrease. This hope still remains farfetched in resource limited settings where albeit the highest global HIV burden [5] HAART still relies on first line treatment because new generation drugs are still very expensive to be afforded by most national HIV control programs. More so, the inexistence of adequate drug toxicity monitoring and reporting schemes [6], underestimates the burden of HAART associated ADRs. In Cameroon, where 5.6% [5] of the adult population is HIV-infected, about 440,000 of whom are eligible for HAART; current HAART coverage is at 58% [7]. Even though a national strategic plan of increasing this coverage to over 75% by 2010 [8] was defined, the issue on the use of new generation drugs was not addressed. This raises an impending concern on future management of treatment associated morbidities. We therefore decided to determine the prevalence of ADRs in patients on HAART at the Douala General Hospital, Cameroon, describe the commonly reported types of ADRs and evaluate their impact on treatment. Our expected findings might help establish clinical guidelines that emphasise on drug toxicity profile as a major criterion for choosing HAART drugs.

## Methods

### Study population and setting

We carried out a hospital-based descriptive cross sectional study at the HIV outpatient clinics of the Internal Medicine unit of the Douala General Hospital, a tertiary healthcare institution with a capacity of 320 beds situated in Douala, the second largest city and economic capital of Cameroon. The study period spanned January 2003 and December 2009. We included in the study, all adult HIV infected patients who for at least six months, had been on a fixed dose combination (FDC) of HAART made up at least three drugs according to the guidelines of Cameroon National AIDS Control Committee. All patients who were on single or two drug antiretroviral therapy for prevention of mother to child transmission during the study period were excluded. Prior to commencing the study, local ethical approval from the hospital was sought.

### Data collection and diagnosis of adverse drug reactions

From the clinical charts of eligible patients, we collected socio-demographic and clinical information including age, sex, weight, start date of HAART, type of HAART regimen, type of reported ADRs, their date of onset, and caregiver decision on whether to maintain or change treatment regimen following ADRs. All treatment modifications due to virological or immunologic failure were not considered. We diagnosed ADR based on patient complaints and/or morphological changes noticed by physicians during routine clinical exam. Prior to commencing HAART at the HIV clinic of the Douala General hospital all patients are counseled on the treatment regimen and potential HAART related adverse effects and they are encouraged to declare them. An adverse effect was considered to be associated to HAART if it was absent prior to HAART and to which other causes could not identified. Most ADRs were diagnosed clinically. Lipodystrophy was diagnosed based on morphological changes like increased breast size, thinning of extremities, excess weight gain and fat redistribution reported by the patient or their family/friends or observed by the physician. Peripheral neuropathy was based on patient's complaint of pain or tingling sensations or pins and needles on the legs and/or hands and/or the presence of abnormal reflexes or abnormal perception of vibrations using a tuning fork. Skin reactions were also diagnosed clinically. The only ADR which was diagnosed both clinically and paraclinically was anaemia.

### Analysis procedure

For data analysis, we used STATA 11.2 statistical software package. The main outcome of interest was ADR. Prevalence of ADR which represented the proportion of the study population who reported at least one ADR was expressed in percentages together with their 95% Confidence intervals (CI). By univariate analysis they were then stratified by other covariates including sex, age, weight and HAART regimen using Mantel Haenzsel analysis. Odd ratios (OR) and their 95% CI were reported. Chi square test was used in contingency tables and a two tailed p value with a significance level of <0.05 was used.

## Results

### Study population

A total of 339 files were eligible during the study period, of which 60.2% (204) were women. Their ages ranged from 17 to 72 years with a median age of 47 years for men (interquartile range (IQR) 41 – 55 years) and 40.5 years for women (IQR 35 – 47). Median duration of HAART was 36 months (IQR: 9 – 68). The median CD4 count was 167cells/µL (IQR: 51 – 265). According to National HIV control guidelines, 5% (17) of patients were on second line FDC HAART regimen and 95% (322) were first line. First line drugs included Nevirapine (NVP), Lamivudine (3TC), Effavirenz (EFV), Stavudine (D4T) and Zidovudine (AZT). 30.1% (102) of our patients were on a combination of AZT-3TC-NVP, 27.73% (94) on AZT-3TC-EFV, 29.20% (99) on D4T-3TC-NVP and 8% (27) on D4T-3TC-EFV (Table 1). Nearly all the patients, 322 (95%) on first line treatment had 3TC in their regimen. More women than men (60.8% vs. 39.0%) were on a regimen containing both AZT and NVP.

**Table 1:** Prevalence of adverse drug reactions (ADR) according to age, sex, and weight and HAART regimen

	N	ADR% prevalence (95% CI)	Odd ratio (95% CI)	P-value
Age group, years				
<25	4	25.0 (24.2 – 74.0)	1.0	†p=0.86
25 – 34	53	20.7 (9.7 – 31.8)	0.8 (0.1 – 8.3)	
35 – 44	133	17.3 (10.8 -23.8)	0.6 (0.1 – 6.3)	
45 – 54	81	18.5 (10.0 – 27.1)	0.7 (0.1 – 7.0)	
>55	68	23.5 (13.3 – 33.7)	0.9 (0.1 – 9.5)	
**Sex**				
Male	135	16.3 (10.0 – 22.6)	1.0	P=0.23
Female	204	21.6 (15.9 – 27.2)	1.4 (0.8 – 2.4)	
**Weight (kg)**				
<60	100	22.0 (13.8 – 30.2)	1.0	P =0.4
>60	239	18.4 (13.5 – 23.4)	0.8 (0.5 – 1.4)	
**HAART Regimen**				
AZT-3TC-NVP	102	10.8 (4.7 – 16.9)	1.0	P=0.01
AZT-3TC-EFV	94	16.0 (8.5 – 23.4)	1.6 (0.7 – 3.6)	
D4T-3TC-NVP	99	29.3 (20.3 – 38.3)	3.4 (1.6 – 7.3)	
D4T-3TC-EFV	27	29.6 (12.0 – 47.2)	3.5 (1.2 – 9.8)	
Other	17	17.6 (1.0 – 36.4)	1.8 (0.4 – 7.2)	
**Total**	**339**	**19.5 (15.2 – 23.7)**	**-**	

AZT: Zidovudine, NVP: Nevirapine, EFV: Effavirenz, 3TC: Lamivudine, D4T: Stavudine.

† P-value for trend

### Adverse drug reactions

A total of 19.5% (n=66) of the study population reported ADRs (Table 1). Age was not associated with ADRs (OR=1.1, 95%CI 0.8 – 1.4, p=0.6). Though women reported more ADRs than men (21.6% vs. 16.3%), sex was not found to be associated with ADRs (OR=1.4, 95% CI 0.8 – 2.4, p=0.23).

**Figure 1:**
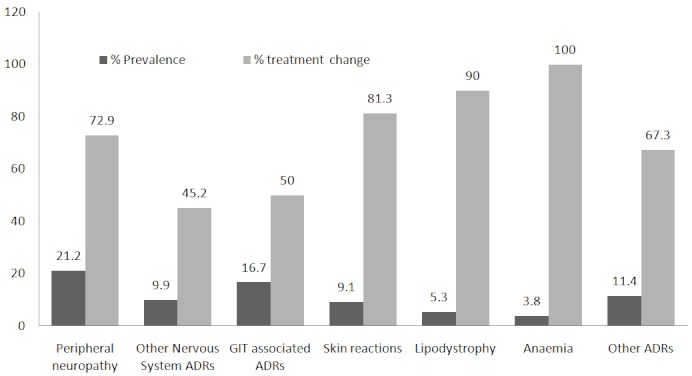
Prevalence of commonly reported adverse drug reactions (ADRs) and their impact on treatment change

The most commonly reported ADR was peripheral neuropathy: 21.2%, (Figure 1) whose median onset was 9 months (IQR: 4.5 – 13.5). Other nervous system associated side effects which included headaches, dizziness, tinnitus and insomnia were present in 9.9% of patients. Gastrointestinal (GIT) side effects were present in 16.7% of patients and had a median onset of 6 months (IQR: 1 – 13). Lipodystrophy accounted for 5.3% of all ADRs (Figure 1) and had a median onset of 23 months (IQR: 13 – 49). Hematological side effects represented 3.8%, the most common being anaemia with a median onset of 5 months (IQR: 1 – 12). The regimen containing D4T-3TC-EFV alone was responsible for 29.6% of ADRs and D4T containing regimens were responsible for 56.1% of all ADRs. Patients who were on D4T containing regimens were more likely to report ADRs than those on other regimens (OR=3.5, 95% CI 1.2 – 9.8, p=0.01) (Table 1). AZT containing regimens were responsible for 39.4% of ADRs.

Among the 66 patients who had ADRs, treatment regimens had to be changed for 68.2% (95%CI 56.8 – 79.5) (Table 2) compared to 31.8% (95%CI 20.5 – 43.2) for whom the same treatment was maintained (p=0.04). Those, whose treatment was maintained, were those in whom ADRs subsided after symptomatic treatment. The regimen containing D4T-3TC-NVP alone was responsible for 53.3% (24) of treatment change compared to other regimens (p<0.001) (Table 2). 71% of all treatment changes were for regimens containing D4T. Lipodystrophy was the main reason for treatment change in 25% of patients. Change of treatment was mostly among women (75.6% vs. 24.4%,p=0.03). No death was attributable to ADRs. Hospital admissions due to ADR were for those who required transfusion because of severe anaemia (hemoglobin

**Table 2:** treatment outcome following adverse drug reactions (ADRs)

HAART Regimen	Treatment changed (%)	Treatment maintained (%)	P-value
AZT-3TC-NVP	6 (13.3)	5 (23.8)	P=0.04
AZT-3TC-EFV	7 (15.6)	8 (38.1)	
D4T-3TC-NVP	24 (53.3)	5 (23.8)	
D4T-3TC-EFV	7 (15.6)	1 (4.8)	
other	1 (2.2)	2 (9.5)	
**Total**	**45 (68.2)**	**21 (31.8)**	

AZT: Zidovudine, D4T: Stavudine

## Discussion

This study was aimed at evaluating the prevalence, describing the commonly found ADRs and their impact on patients taking HAART at the Douala General Hospital. Our findings show that about 1 in every 5 patient (19.5%) on HAART, reported at least one ADR within a minimum period of less than a month. This is consistent with findings in an Indian study of 400 patients on HAART in 2010 where the prevalence of ADRs was 17.5% [9] but markedly lower than what was reported in urban Kenya in 2007 where HAART-related ADRs were present in 40.6% of patients [10]. This difference may be explained by the lack of uniformity in the reporting style of ADRs across settings [6] even though all of the patients in these settings are on similar FDC generic drugs. However, regional or ethnic susceptibilities to ADRs might also explain this difference. In our study population, more than half of all ADRs were reported in patients taking regimens containing D4T and the most common was peripheral neuropathy (21.2%). Our findings are similar to those in other African settings like Kenya where in an urban population in 2007, the prevalence of peripheral neuropathy was 20.7% [10], and in a rural Ugandan population in 2007 it was found to be 17.2% [11]. However, it is lower than findings in 2005 in Botswana where peripheral neuropathy was found to account for 35% of all ADRs [12]. These differences might be explained by the fact that the Botswana study was a randomised clinical trial in which all patients on HAART were intensely screened in search of ADRs whereas in our cross sectional study, ADRs were mostly self-reported. We found that about three quarters of patient with peripheral neuropathy had their treatment changed which resulted to a relative amelioration but not total disappearance of their symptoms. This finding is different from that of an Indian study in 2010 [9] where the author found that discontinuation of Stavudine was associated with total recovery. However, other causes of peripheral neuropathy like distal symmetric polyneuropathy [13] which could have been asymptomatic in our patients prior to HAART and revealed by HAART could explain why symptoms persisted.

Lipodystrophy occurred in 5.3% of those with ADRs. Chang et al in 2002 found a prevalence of 3.5% among Korean patients on HAART [14]. The prevalence of Lipodystrophy varies considerably across studies: in a Botswana study of 650 adults in 2007 Lipodystrophy was 16% of all ADRs [15], 34.2% in a Rwandan population of 409 adults in 2007 [16]. Lipodystrophy occurred more frequently (86.7%) in patients who were on a regimen containing D4T-3TC-NVP and patients on a D4T regimen were more likely to develop ADRs than those not on D4T which is similar to what Van Griensven et al. found in 2007 where they reported that Rwandan patients on D4T were three times more likely to develop Lipodystrophy compared to those on AZT [16]. Lipodystrophy was the reason for treatment change in 1 of every 4patients with ADRs.

Hematological ADRs represented 3.8% of all ADRs of which anaemia (haemoglobin <7g/dl) was the most common and the most severe, all of which were associated to AZT-containing regimens. This is slightly higher than what Bourgeois et al. found in Yaoundé, Cameroon in 2005 where AZT-related severe anaemia represented 2.8% of all ADRs. Our findings are similar to that of Ivory Coast of 3.4% in 2006 [17], but lower than that of a Malawian study in 2002 [18], and a Senegalese study in 2007 [19] where AZT-related anaemia were 7.8% and 6.3% respectively. However, Forna et al. in rural Uganda in 2007 found 0.4% [11]. Nevertheless, these differences might reflect the relative use of AZT containing regimens in different settings. When we correlate the median time of onset of anaemia to the fact that baseline (pre-HAART) hemoglobin of >10g/dl in our setting is a prerequisite to initiating an AZT-containing regimen, we can conclude that anaemia in these patients is associated to AZT [20] most espeically as AZT discontinuation was also associated with improvement of Hemoglobin levels even in patients who were not transfused. This is consistent with findings by Koduri et al. [21] in a clinical case report of three patients with severe AZT associated anaemia who recovered after AZT was stopped. All our patients with anaemia had their treatment changed similar to what was found in an Iranian study in 2009 where haematological ADRs were among the most common causes of treatment discontinuation [22]. Nevertheless AZT-associated ADRs are likely to persist as a cause of HAART-associated morbidity in Cameroon, firstly because almost two thirds of our study population was an AZT-containing regimen and secondly because AZT still remains a main first line drug in HAART especially for the prevention of mother to child transmission of HIV.

GIT associated ADRs had a prevalence of 16.7%. Their occurrences in other African settings vary enormously: 1% in Kenya [10], 38% in Malawi [23]. Although HAART-associated ADR were common, none were fatal. This finding is similar to that of a South African longitudinal study where no fatal cases of ADRs were recorded in 665 in-hospital patients, 6.3% of whom were admitted as a result of HAART associated ADRs and 6.3% others who developed HAART associated ADRs in hospital [24].

## Conclusion

This small descriptive study, whilst informative in the context of Cameroon has a number of limitations. Firstly, the fact that data was collected from clinical charts is a source of reporting bias. Secondly, the fact that most ADRs were self-reported and not investigated by laboratory tests, might have led to over-representation of some and under-estimation of others which might have been detectable paraclinically. That notwithstanding, ADRs associated with HAART are common in Cameroon. Therefore, caregivers should actively look for them because they are a potential threat to the effectiveness HAART. Patients education on HAART associated ADRs should be part of the HIV care package so as to facilitate reporting and management. Stavudine containing regimens appear to elicit the highest proportion and the most serious ADRs so should no longer be part of current therapy. Finally, the introduction of newer generation drugs with low toxicity in resource constrained settings is mandatory so as to ensure the provision of effective quality care to people living with HIV.
